# Observational study on the non-linear response of dolphins to the presence of vessels

**DOI:** 10.1038/s41598-024-56654-6

**Published:** 2024-03-13

**Authors:** Roee Diamant, Alberto Testolin, Ilan Shachar, Ori Galili, Aviad Scheinin

**Affiliations:** 1 Department of Marine Technologies, University of Haifa, Haifa, 3498838 Israel; 2 Faculty of Electrical and Computing Engineering, University of Zagreb, Zagreb, Croatia; 3 Department of Mathematics and the Department of General Psychology, University of Padova, 35131 Padova, Italy; 4 Morris Kahn Marine Research Station, Department of Marine Biology, Leon H. Charney School of Marine Sciences, University of Haifa, Haifa, Israel

**Keywords:** Impact of underwater radiated noise, Dolphin whistles, Dolphin classification, Signal detection, Whistles clustering, Behavioural ecology, Signal processing, Marine biology

## Abstract

With the large increase in human marine activity, our seas have become populated with vessels that can be overheard from distances of even 20 km. Prior investigations showed that such a dense presence of vessels impacts the behaviour of marine animals, and in particular dolphins. While previous explorations were based on a linear observation for changes in the features of dolphin whistles, in this work we examine non-linear responses of bottlenose dolphins (*Tursiops Truncatus*) to the presence of vessels. We explored the response of dolphins to vessels by continuously recording acoustic data using two long-term acoustic recorders deployed near a shipping lane and a dolphin habitat in Eilat, Israel. Using deep learning methods we detected a large number of 50,000 whistles, which were clustered to associate whistle traces and to characterize their features to discriminate vocalizations of dolphins: both structure and quantities. Using a non-linear classifier, the whistles were categorized into two classes representing the presence or absence of a nearby vessel. Although our database does not show linear observable change in the features of the whistles, we obtained true positive and true negative rates exceeding 90% accuracy on separate, left-out test sets. We argue that this success in classification serves as a statistical proof for a non-linear response of dolphins to the presence of vessels.

## Introduction

In this study, we explore the impact of the presence of vessels on the vocalization of bottlenose dolphins. We focus on dolphins due to their occurrence in nearly all tropical and temperate regions, both in coastal and offshore waters. They are apex predators, acting as sentinel species and important bio-indicators. Their wide range of distribution makes them ideal representatives in this study, as they show plasticity in habitat choices and can migrate to another environment in the event of a strong disturbance like sound^1^.

The impact of vessels on marine mammals is a well studied area^2^. For marine mammals, underwater radiated noise (URN) from vessels can impact the individuals in five ways^2,3^: (I) audibility, which depends on the species’s detection threshold; (II) behavioral responses reflected by alterations in the intensity, frequency, and intervals of the individual’s produced sound^4^ and by stress factors; (III) masking of sound-affecting communication, localization, and foraging^5^; (IV) physiological auditory threshold shifts due to fatigue in the hair cells of the inner ear^6^; and (V) physical damage (injury) to the auditory system^7^. As a result, standards were set to limit the transmitted acoustic power per exposure time^8^. To quantify the impact of vessel noise, a measure for the URN experienced by the dolphins is required. This can be produced by either the attachment of passive acoustic tags to individual dolphins, which is an invasive and ethically controversial technique, or the estimation of the URN by applying acoustic propagation models for the evaluated location of the vessel and dolphin, which requires the setup of a complex acoustic array and an accurate measurement of the local bathymetry and sound speed profile. Instead, in this study we avoided characterizing the vessels’ URN and its effects on the dolphins, and rather explored the response of the dolphins to the presence of vessels.

Previous studies have explored the effect of the presence of vessels on dolphins. Here, we review the main methodologies. One recent work analysed the differences in the dolphins’ whistles emission rate and signal duration in the presence of vessel transit routes^9^. Roughly 1800 whistles were compared before, during, and after transit occurrences, and average differences were observed during and immediately after the passing of nearby vessels. A similar conclusion was drawn in another recent study that investigated the impact on whistle parameters due to vessel approach and engine shutdown^10^. The method involved a target vessel that turned its engine on and off, and the analysis compared features of approximately 100 recorded whistles at intervals of 0–5 min and 5–10 min after engine shutdown. For dolphins of the oceanic ecotype, results showed differences in the peak frequency and in the whistle rate, while no change was observed for dolphins of the coastal ecotype. A possible conclusion drawn was that coastal dolphins are more acclimatized to vessels’ presence. Supporting this conclusion are differences in the whistle band frequency between the oceanic and coastal groups, as analyzed in another interesting case study focused on the response of dolphins to the significant reduction in shipping activity during Covid‘19^11^ and in a recent Ph.D. thesis that analyzed differences in the vocalization of white-beaked dolphins in Iceland during Covid-19^12^. Using AIS records, the authors found roughly 15 k indications of vessel presence in 2020, compared to nearly 30 k in 2022. Analysis of continuous recordings over several months for both years showed that whistle rate was higher by roughly 20% in 2020. The spectral characteristics of whistles while distinguishing between the presence and absence of tour boats was the subject of yet another recent Ph.D. thesis^13^. While adrift next to a group of dolphins, continuous recording of the dolphins vocalizations were recorded during occurrences of approaching vessels. Analysis of the histograms of high signal to noise ratio (SNR) from roughly 150 whistles showed differences in the minimum, maximum and starting frequency of the whistles, which were explained as a possible strategy to avoid increase of energy expenditure. A longer-term analysis compared between two sites of different vessel activity^14^. Results for roughly 1000 whistles showed statistically significant differences between the distribution of whistle features from the two groups. More specifically, a higher number of whistles was detected in the site where vessel presence was more continuous. In the same site, an increase in the signature of the dolphins whistle modulation was observed.

While the above works describe changes in whistle waveforms in the presence of vessels, we argue that some gaps exist in the available methodological tools. In particular, to evaluate impact of the presence of vessels in cases where the features of the dolphin’s whistle do not follow a simple correlation with presence indicators of vessels. In other words, when there is no linear relation between the whistle features and vessel presence. For example, while focusing on the echolocation clicks of finless porpoises, recent work showed that, in complex environmental conditions, the acoustic response of the individuals to vessel presence is not linear^15^ and the signal features show fluctuations in an attempt to adapt to the changing noise level. A step towards a non-linear exploration was offered in a previous study that instead of examining the values of the features explored the variability in the whistle features as a function of environmental parameters^16^. Analysis of a total of roughly 2000 whistles from 60 different dolphin groups revealed clear association between signal features variability and vessel presence. Still, the method involves a linear analysis in the form of a principle component analysis (PCA) which associates a cause with the observed variability, and thus avoids the exploration of other non-linear relations. Moreover, especially when the the whistle feature values cannot be well separated between cases of vessel absence or presence, it is hard to uniquely associate a cause through an increase in variability, and there is a need to quantify non-linear impacts. Such a non-linear evaluation requires the analysis of a sufficiently large dataset from a singular site and for a specific group of dolphins. In this research, we use non-linear classification tools to process roughly 50,000 detected whistles, and show evidence for non-linear relations.

To address the goal of exploring complex dolphin response to vessel presence, in this work we classified non-linear differences in the vocalization of dolphins. Dolphins are known to express stress through acoustic behaviour. For example, the signature-whistle rates were shown to be the most telling vocal indicator for short term stress when dolphins in Sarasota Bay (Florida) experienced capture-release events compared to undisturbed conditions^17^. Other work^18^ showed that signature whistles comprised 50% of the vocal repertoire of free-ranging bottlenose dolphins as opposed to 90% in temporarily restrained dolphins. The study also found that the number of loops in a whistle increased during capture-release conditions compared to undisturbed conditions. Differences in the structure and rate of dolphins whistles can thus serve as a classification metric to distinguish between two cases: when a vessel is present and when there is no vessel around.

Our research hypothesis is that, if affected by vessel presence, the whistles of the dolphins can be used to predict vessel presence. This prediction involves the binary classification of features from detected dolphins whistles into a “With vessel” class, indicating that a vessel is present, and a “No vessel” class, indicating that no vessel is around. Consequentially, the analysis results are explored in terms of the classification confusion matrix. In particular, the true positive and the true negative; that is, to show if the classification attempt indeed succeeds. We take this approach since a classifier may capture non-linear relations, which are otherwise hard to distinguish. We argue that a statistically significant success in such prediction would prove that dolphins respond to the presence of vessels. While we do not make an argument about the cause of this reaction, may it be a sign of stress or notification of vessel presence, we explore features in the structure of the dolphin whistles and quantities that indicate such a response. We further analyze what are the dominant whistle features in such events, and use this analysis to comment on the meaning of the impact of vessel presence.

To remove possible biases in the results due to different dolphin populations and sea conditions, we offer a statistical analysis for responses over a specific population of bottlenose dolphins. Alongside visual aid verifying the existence of the same dolphin population, we explored dolphins’ responses to vessel presence from opportunistic vocalizations identified within a large dataset of continuous passive recordings. In particular, we performed long-term acoustic recordings in a marine protected area close to both a known dolphin habitat and a shipping lane, to serve as a proxy for behavioral implications. We identified a large number of vessels present in the recording period by detecting URN of vessels (We note that reliable AIS recordings were not available in the test site) and a large number of dolphin whistles within the recorded database, and aimed to statistically explore if the dolphins responded to vessel presence.

## Results

### Preliminaries

To explain the results below, we will first provide a description of our testbed, the content of the database, and a short introduction of the analytical methods. A more through description of the methods then follows in the “Methods" Section.

**Figure 1:**
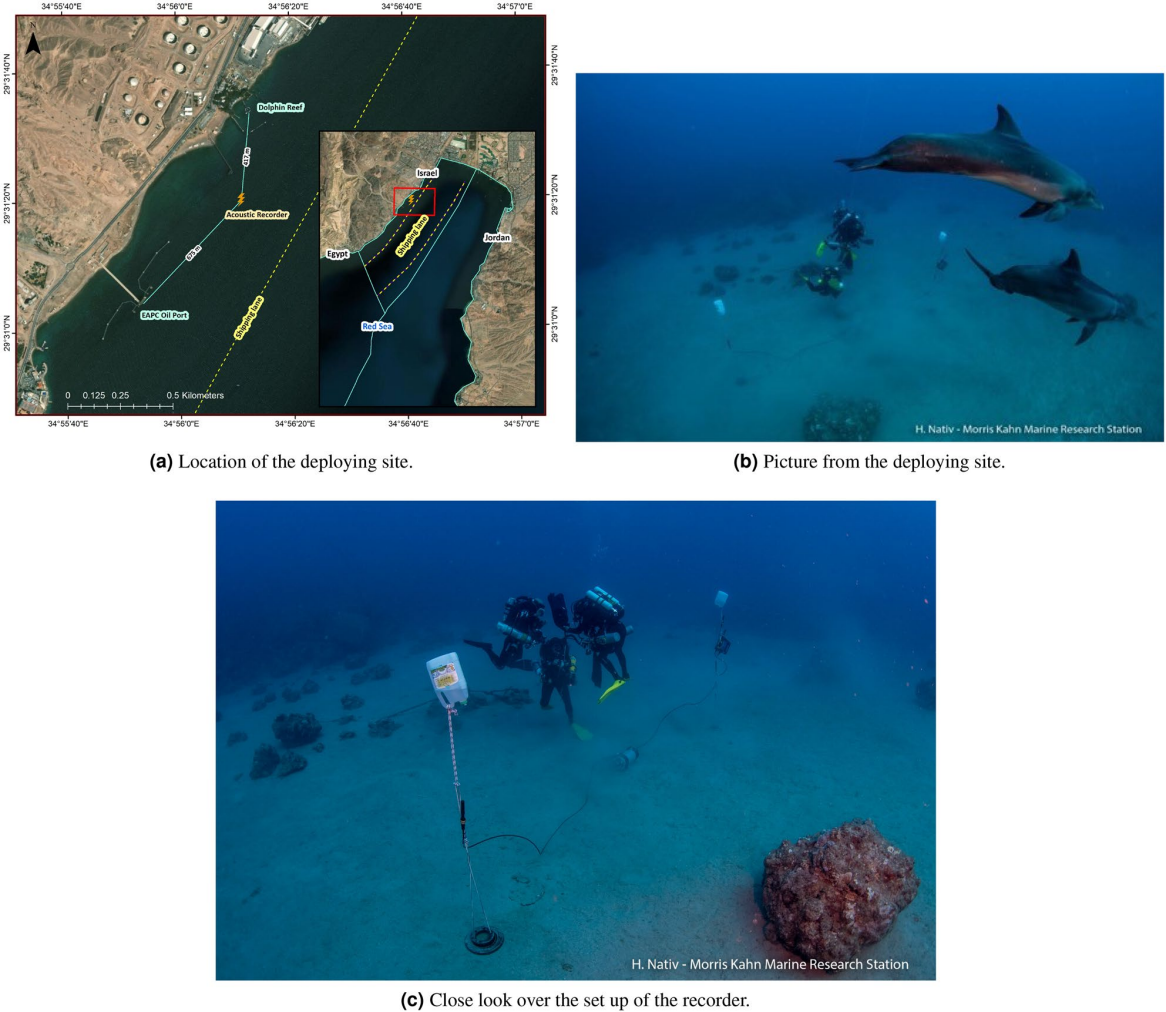
A map of the deployment site (vessel shipping lane is marked in yellow line and deployment location is marked in orange), and two pictures of the deployment setup with dolphins inspecting the instruments. Eilat, July 2021. The two white bottles are floats set to lift the hydrophones 1 m above the seabed.

#### Description of the testbed

The research was conducted in the vicinity of the ’Dolphin Reef’ facility, in the Red Sea of Eilat, Israel, see map in Fig. 1a. At the time, the ’Dolphin Reef’ was home to four captive bottlenose dolphins (*Tursiops truncatus*), three mature females and a young male, that had spent their entire life in human care, though they frequently swam in the open sea, as the ‘Dolphin Reef’ boundaries were not enclosed by fences or nets. Since the average group size of bottlenose dolphins is five individuals^19^, we argue that four individuals is a good proxy to an average group. To verify that during our data collection only these four dolphins were present in the vicinity of the recorder we relied on reports from park rangers and inquiries of visitors to the marine protected area, but also on designated visual observations. These observations took place for three days in the beginning of the recording period, one day in the middle of the recording period and two additional days towards the end of the recording period, and lasted between 3 and 5 hours per day. The observer stood on a dock some 10 m above the water and had a clear view over at least 5 km north and south of the recording location, and 2 km west from the recording location. Visual inspections monitored both dolphin occurrence and vessel activities.

The adjacent coastal property is the Europe Asia Pipeline Company (EAPC) Oil Port, which receives oil imports from large shipping vessels that dock at a designated pier, located 1 km from the ‘Dolphin Reef’ facility. A commercial shipping port and navy port are also a few kilometers up the coast, and all large vessels associated with these assets introduce much noise disturbance to the marine environment in the waters surrounding the ‘Dolphin Reef’ facility. In addition to shipping vessels, many small recreational motor vessels frequent the area, and often approach the ‘Dolphin Reef’ boundaries to observe the dolphins. All large commercial vessels pass in a 2 km corridor whose western boundary is located only 300 m from the underwater acoustic recorder deployment site. This area was thus ideal for the study as it provided a combination of acoustic disturbance from both small and large vessels, and guaranteed consistent dolphin presence, of the same four individuals, who would present a similar vocal responses across the entire study period. The recording device was placed on the sea-floor at a depth of 60 m, at location 29^∘^31’ 19.9” N 34^∘^56’ 11.0” E, at a distance of approximately 500 m from the ‘Dolphin Reef’ and 600 m from the EAPC Oil Port pier.

Our testbed included two self-made acoustic recorders, each of which included two GeoSpectrm M36 hydrophones, a pre-amplifier, a sampling card, a processing unit, and a battery large enough to last for two months of data collection. The electrical components were mounted in a custom designed underwater casing with two underwater cables leading to the hydrophone units. Before deployment, the acoustic sensitivity of the entire system was measured in an acoustic tank using a calibrated sound source, and the results showed a flat frequency response with less than 1 dB across the 5–20 kHz band which fits most of dolphin’s whistles^17^. The units where anchored such that the hydrophones were hovering 1 m above the seabed. The hydrophones were set 5 m apart for low correlation between the two channels, and the recorders were placed on patches of sand to reduce self-noise (Fig. 1b). Our testbed also included an automatic identification system (AIS) receiver that collected AIS transmissions of passing vessels. The AIS receiver was stationed on the top of a building located roughly 1 km from the deployment site. It was able to pick up vessel signals up to a distance of 50 km. However, visual identification of vessels proved that the AIS information is biased, as most vessels did not operate their AIS, and thus vessel tagging was performed based on the recorded acoustic data. We recorded raw acoustic signals for a period of 22 days during the month of June 2021. During this period, we used a local weather station to track wind intensity, wave height and water temperature. We note that, as customary for the Red sea of Eilat, the weather conditions were extremely stable with wave height not exceeding 0.5 m, and water temperature remaining between $24^\circ$ and $25^\circ$. No rain occurred during the data collection period.

#### Description of the datbase

**Figure 2:**
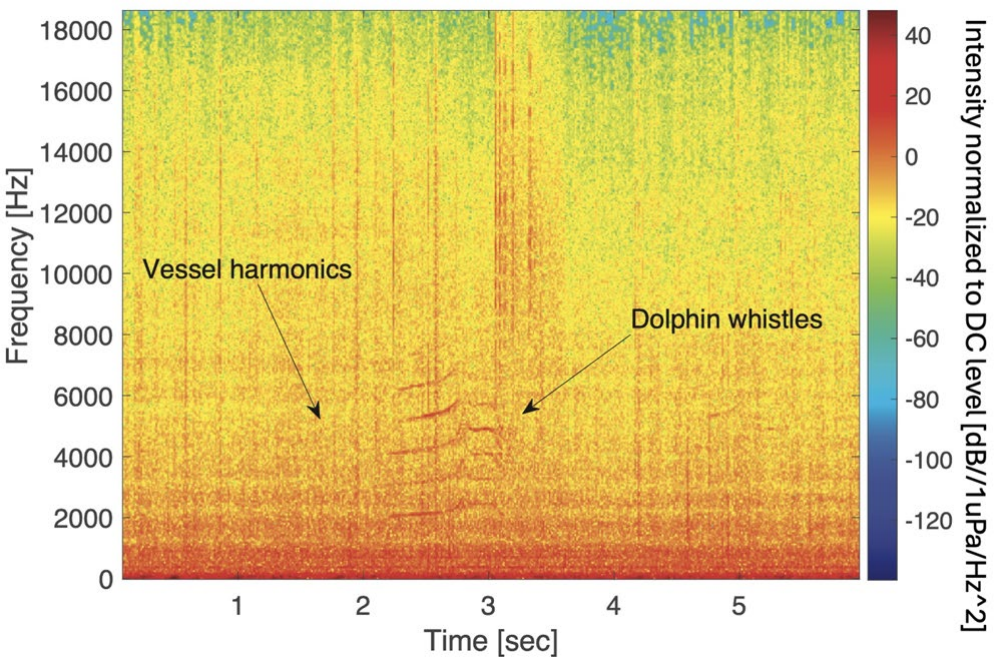
Spectrogram showing dolphin whistles masked by radiated noise from a container ship recorded in the Red Sea, June 2021. The red color indicates higher acoustic intensity.

The collected data were sampled at 96 kHz at 3 bytes per sample, and was fragmented into short files of 300 s from which time-frequency spectrogram matrices are formed. An example of such spectrogram comprising of both dolphins’ whistles and vessel indication is shown in Fig. 2. We analyzed the recorded data offline in two threads. For vessel identification, manual tagging was performed by an expert, with vessels identified by their spectral signature, as well as by listening to the recorded data. This resulted in a dataset of 7546 identified vessels. Based on the spectral content of the recordings, we identified that roughly 80% of the vessels were large cargo vessels, while also some recreational vessels were spotted.

For dolphin identification, we used a machine learning (ML)-based procedure. To train the ML detector, we engaged high-school students from the ’Open School’ in Haifa, Israel to manually annotate part of the dataset (see details in the “Methods" section). The results from the 22 days of data collection were 82,340 identified whistles. Out of these, we selected the 55,852 whistles whose SNR exceeded 15 dB. This filtering reduces errors in the process of feature extraction, which is sensitive to low SNR, especially for harmonic identification, clustering and duration estimation.

Based on the identified vessels, we classified dolphin whistles into those emitted in the presence of vessel URN, termed *with vessel*, and whistles detected when no URN from a vessel was present, termed *no vessel*. The former is defined by vessel presence within a time buffer of 1 min before or after the dolphin whistles. The latter is determined if no vessel was identified within a time window of 5 min before and after the whistle detection event. Other whistles were excluded from analysis. Vessel identifications were obtained by manually observing the specgrogram of each time buffer. In particular, a sonar expert looked for vessel acoustic features: harmonics of vessel engines; increase in the broadband noise intensity; or the presence of narrowband modulations of a thruster. Indications of vessel presence were verified by a second expert. This resulted in 25,982 whistles labeled as ”vessel”, and 25,249 whistles labeled as ”no vessel”.

After identifying and classifying the dolphin whistles, the data were further processed to extract features. We chose features recognized as behavioral indicators^20,21^, as well as behavioral stress indicators^22,23^. The feature set included whistle duration (*duration*), where, since the duration of harmonics and multipath signals may appear shorter or longer due to lower SNR or temporal spreading, respectively, we consider the time period from the beginning of the whistle to its end with no averaging performed; the number of basic whistles, i.e., no harmonics or multi-path, in a time buffer of 60 s (*whistle number*); the number of whistles overlapping with the identified one (*number of overlaps*); the number of traces that reflect harmonics and multi-path of a single whistle (*number of cluster*); and the harmonic spectral rate of the whistle (*harmonic rate*), where, we measure the ratio by which the frequency of the harmonic changes from the basic whistle structure, normalized by the harmonic number, averaged over the harmonic duration and over all harmonics. We clarify that, while qualifying as one of the characteristics of the channel, we included the multi-path in the ’number of cluster’ feature since more multipath arrivals are expected to pass the detection threshold the louder the whistle is. We report that other tested features did not yield good classification results and were thus not incorporated in the final analysis. These include the whistle bandwidth, the maximum and minimum frequency, a 6-degree polynomial representation of the whistle time-frequency trace, symmetrical shape characteristics, and the variance of the signal strength across the bandwidth. We note that absolute signal strength was not considered since we could not verify the location of the dolphins and therefore their distance from the underwater recorder.

### Classification results

**Figure 3:**
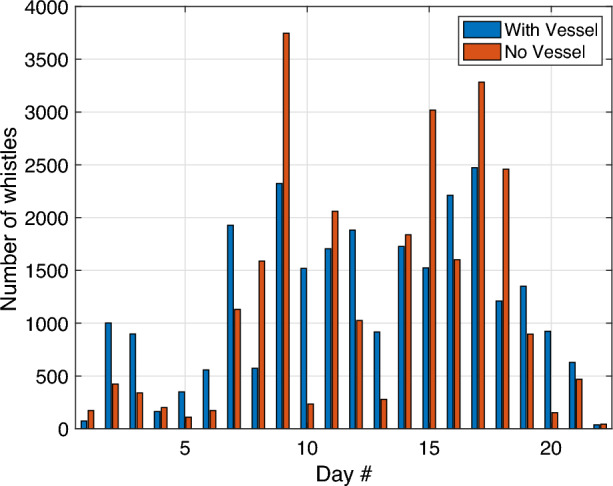
Number of detected whistles with and without the presence of nearby vessels.Data are divided into “with vessel” whistles (blue) and “no vessel” whistles (red).

**Figure 4:**
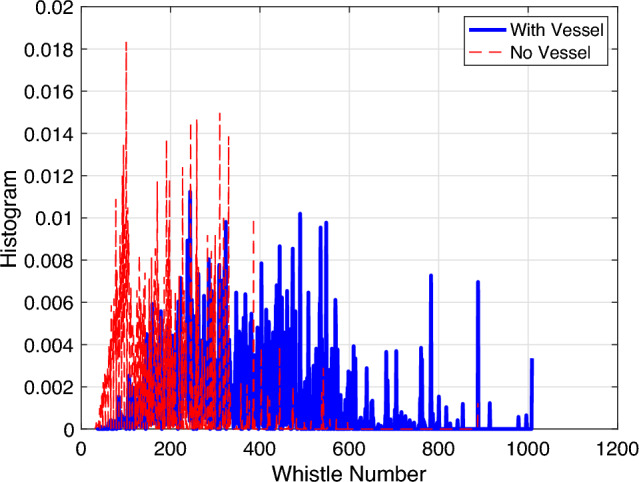
Histogram of the “Whistle Number” feature as collected from in all 22 days. A partial overlap is observed between the “with vessel” and “no vessel” classes.The histograms show similarities between the distributions of the features, and thus rules out the choice of a linear classifier. Data are divided into “with vessel” whistles (blue) and “no vessel” whistles (red).

A histogram for the number of detected dolphin’s whistles per recording day is presented in Fig. 3. We observed a notable difference in the number of detected whistles across different study days. We therefore considered whistles acquired in different calendar days as independent. We also observed that more than 1000 whistles were detected during most days, which allows for reliable statistical analysis. From the raw data, no apparent difference were shown between the number of “with vessel” and “no vessel” whistles. This is further explored in Fig. 4, where we observed that the ‘Whistle Number’ feature of the “with vessel” class significantly overlaps with that of the “no vessel” class. Interestingly, Fig. 4 also showed that the ‘Whistle Number’ feature of the “with vessel” class is generally higher than that of the “no vessel” class. That is, in-spite of the URN of the vessels, the dolphins tend to produce more vocalizations when a vessel was in the vicinity. This result also proves that, using our methodology, the dolphins’ whistles were identified despite the presence of vessels (i.e., at lower SNR).

**Figure 5:**
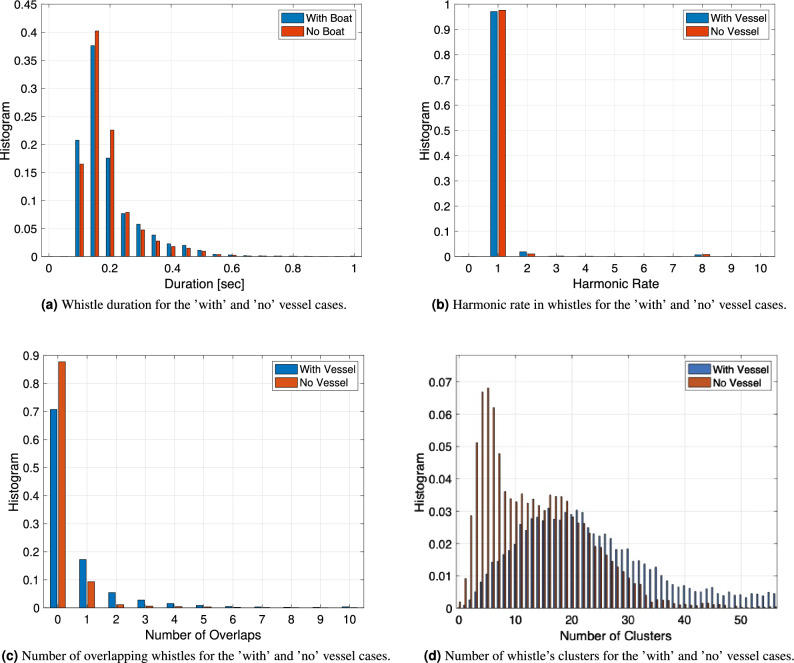
Histogram of features of the whistles with and without vessel. (**a**) ’Duration’. (**b**) ’harmonic Rate’. (**c**) ’Number of Overlaps’. (**d**) ’Number of Clusters’. Data are divided into “with vessel” whistles (blue) and “no vessel” whistles (red).

Histograms of the other four significant whistle features, namely the ‘Duration’, the ‘Harmonic Rate’, the ‘Number of Overlaps’ and the ‘Number of Clusters’ are shown in Fig. 5a–d, respectively. As in the case of the ‘Whistle Number’ feature, in all four cases we observed a significant overlap between the values for the “with vessel” and “no vessel” classes. According to these result we conclude that the relationship between the dolphin’s whistle and the presence of a vessel is not trivial and cannot be detected by means of simple statistical segmentation. We thus chose a support vector machine (SVM) with a non-linear radial basis kernel function as the classification method, whose inputs are the five features of the dolphin whistles.

**Figure 6:**
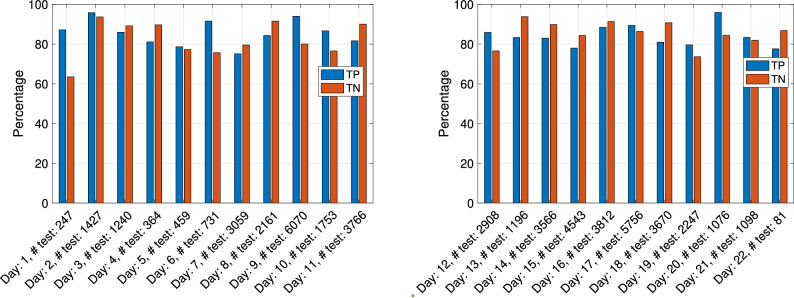
Classification results of dolphin whistles: percentage of correct identification of boat indications (True positive) and percentage of correct identification of no boat indications (True negative). Left panel: days 1–11. Right panel: days 12–22. Data are divided into “with vessel” whistles (blue) and “no vessel” whistles (red).

**Figure 7:**
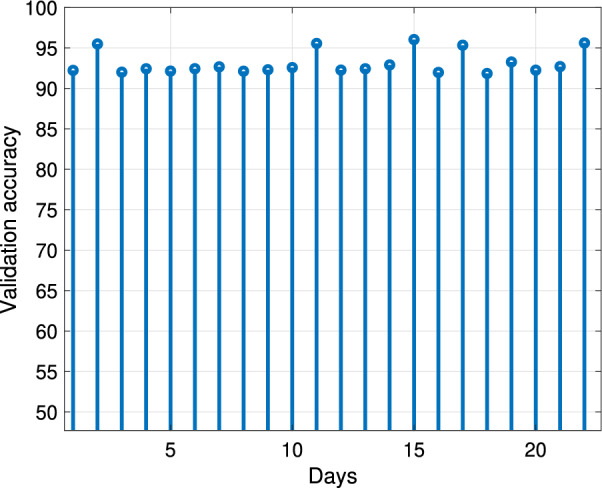
Per day accuracy in classification during the Validation Stage.

**Figure 8:**
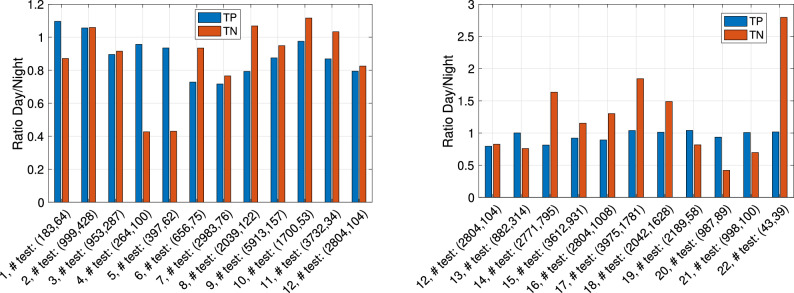
Ratio between classification results of day and night. In brackets number of test whistles: (day,night). Left panel: days 1–11. Right panel: days 12–22. Data are divided into “with vessel” whistles (blue) and “no vessel” whistles (red).

The classification results of the “with vessel” and “no vessel” dolphin whistle classes are shown in Fig. 6 in terms of the true positive (termed TP: correct classification to “with vessel”), and true negative (termed TN: correct classification to “no vessel”) rates. Results are shown separately for each day, considering the corresponding calendar day as testing data. For example, producing results for day *X*, we trained and validated our classifier based on all 51,231 whistles detected except from those whistles detected on day *X*, while testing and calculation of the TP and TN rates was performed only for those whistles detected on day *X*. The number of whistles used for such testing per day is given in the labels of the bars. We observed that, for all days, TP exceeds 75% and is 84.8% on average. The TN rates are slightly lower on average, reaching 83.9%, and exceeding 73% for all days but Day 1, where the TN is 63%. The stability of the results across calendar days demonstrates the generalization (i.e., robustness) of the classification, which is also evident when observing the stable validation accuracy in Fig. 7, where, as in Fig. 6, the data for validation is considered per calendar day. As expected, the validation accuracy is better than the classification accuracy measured on the test set, further suggesting that there was no risk of overfitting. These classification results, which are much higher than chance level, demonstrate the existence of a strong relation between the dolphin whistles and the presence of a vessel, though not a trivial one. To explore differences between daytime and nighttime activity, in Fig. 8 we show the ratio between the TP and TN results obtained for whistles detected during the day and during the night. The total number of detected whistles was 42,926 and 8305 for daytime and nighttime, respectively, which is not balanced. Also the vessel activity during nighttime was roughly 10% of that during daytime. Still, the number of defections at nighttime was high enough to perform classification. The results show that, for most days, the difference in TP and TN between daytime and nighttime is small, with an average daytime versus nighttime ratio of 92% for the TP and 106% for the TN.

**Figure 9:**
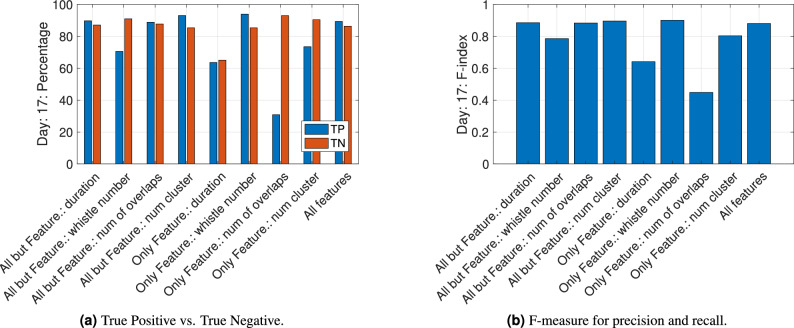
Impact of the 5 considered features. Results shown for day 17 (5756 whistles). Data are divided into “with vessel” whistles (blue) and “no vessel” whistles (red).

To better understand the importance of different whistle features on the vessel classification, we repeated the process using different feature spaces. For example, to explore the impact of feature *i*, we performed the classification using all features except feature *i*, and another classification using only feature *i*. The results are then compared with the TP and TN obtained when using all features. To prove this point we show in Fig. 9 testing results from Day 17, which includes the largest amount of whistles. We note that similar results were obtained for other calendar days. We observe that, in terms of the TP, TN, and the F-measure, classification accuracy when using only the ‘Whistle Number’ feature does not deteriorate much compared to using all features, while classification results are significantly impacted when removing this feature from the dataset. We therefore conclude that this is the most important feature for discriminating the presence of nearby vessels.

Finally, we examine changes in the communication rate of the dolphins. This cannot be observed directly based on the number of detected dolphin whistles per recording day (as in Fig. 3), but rather based on the features related to the communication rate, namely the ‘Whistle Number’, the ‘Number of Overlaps’ and the ‘Number of Clusters’. To quantify this, denote $\rho (i)$ as the ratio between the mean of feature *i* for “with vessel” whistles and its mean for “no vessel” whistles. We report that for the above features related to the communication rate, the value of $\rho$ is 2.13, 2.80, and 2.81, respectively, whereas for the ‘Harmonic Rate’ and the ‘Duration’ features, which are less related to the amount of communication sessions, we obtain $\rho =0.99, 1.02$, respectively.

## Discussion

Our results in Fig. 4 and in and Fig. 5d show that there is no linear relation between the whistle features and the presence of vessels, and that the values of the features overlap for the two cases of ’with’ and ’without’ a vessel presence. To find if a more complex relation exists, we turned to non-linear classification where, rather than exploring linear effects such as if dolphins emitted more or less whistles in the presence of vessels, we predict if a vessel is present using features of a dolphin whistle.

Our results show that different to previous works^9–11,13,14^ that observed differences in the whistle rate when vessels are present, we did not observe such a linear relation in our dataset, and that there is overlap in distributions for the ’with’ and ’without’ vessel cases for each of the whistle features. While one possible explanation is that our results are specific for the four explored dolphins, the differences could also be explained by the high number of whistles and vessels explored, which may capture more complexities within the dataset. Still, generalization of our results is difficult as dolphins vocalize differently in different marine environments. In the following, we thus refer only to the community of dolphins we explored and to the methodology used. Due to the non-linear relation between the whistle features and the presence of vessels, we cannot conclude on the actual change in the whistle when a vessel is present. Still, observing the more significant features in the whistles that contributed to the success of the classification, we can comment about conceptual differences. We conclude that both by the high values of $\rho$ obtain for the three features related to the communication rate, and based on the results in Fig. 9 that show that the ‘Whistle Number’, the ‘Number of Overlaps’, and the ‘Number of Clusters’ features are all important for classification. We thus argue that, in the presence of a vessel, the dolphins’ whistle emissions are clustered together, and the vocalization is denser. Such more dense communication may project on stress or excitement of the dolphins. While these results can be explained by an increase in the number of dolphins present, we argue that, due to the small number of dolphins, such effects are filtered out in the statistical evaluation of our database of $>100$ k whistles. According to the results in Fig. 9, we also observe that, while difference in value are not apparent between the two classes, the ‘Duration’ feature holds information for classification. Since this feature is related to the shape of the whistles, we deduce that the structure of the vocalization also changes when a vessel is present. Following the comparison between daytime and nighttime in Fig. 8, we conclude that the observed changes in the dolphin whistles due to the presence of vessels do not change significantly during the period of the day.

Previous studies have attempted to link dolphin vocalizations with specific behaviors^24,25^, though it is challenging to establish a clear relationship due to the variable interpretation of some behaviors and the fact that they often occur out of sight of observers. In the case of our study, since dolphins are known to express stress through acoustic behaviours^17^, it could simply be an acoustic expression of stress. An alternative explanations could also be: either the presence of vessels ‘activates’ the nearby dolphins and creates a reason to communicate, such as coordination of approach or avoidance, or rather, the noise introduced into the water masks the whistles produced, thereby causing the dolphins to vocalize more in order to assure that they are indeed heard despite the surrounding noise.

The fact that, due to the highly complex acoustic propagation pattern and the limitations of our setup, we could not evaluate vessel URN intensity as experienced by the dolphins, limits our conclusions to a broad effect of vessel presence rather than to vessel URN level. A consequence of this limitation is that the response of the dolphins could not be associated with vessel URN characteristics, vessel URN intensity or vessel distance from dolphins. Further, without the ability to localize the dolphins, we could not identify how many individuals were emitting whistles at a given time window, and thus could not be entirely certain if the effects shown are due to a change in the vocalizations of the dolphins. However, we argue that the large quantity of whistles detected reduces this ambiguity. The results collected apply to the small population of the four dolphins in the ’Dolphin Reef’ facility, which are likely acclimatized to vessel URN while swimming beyond the facility boundaries, since the area explored is dense in vessel activity. Here, we argue that, if this is the case, then no response from the dolphins was expected. Overall, it appears that vessel acoustic disturbance strongly affects dolphin whistle vocalization, though it is not possible to quantify its hindrance to the dolphins. We can still claim that vessel presence seems a main factor of disturbance, and should be monitored similarly to a pollution factor.

Future work should further explore the influence of vessel noise on dolphins, for example by labeling vessels according to their acoustic signature, which would allow to investigate whether the responses of the dolphins change according different types of vessels (e.g., large ships vs. recreational boats). Further questions to explore could also be, for example, whether the response of the dolphins to vessels becomes more evident as the vessel noise experienced by the dolphins increases, an analysis which requires an estimate of the dolphin and vessel locations, or whether it is evident only for a specific type of noise. However, such studies would need to take place in areas where, unlike Eilat, AIS transmissions are mandatory. Other interesting research directions would be to repeat the same analysis for different populations of dolphins, or to record spatial information which would allow a study on how behavioral changes are related to the proximity between the dolphin and the vessels.

## Methods

**Figure 10:**
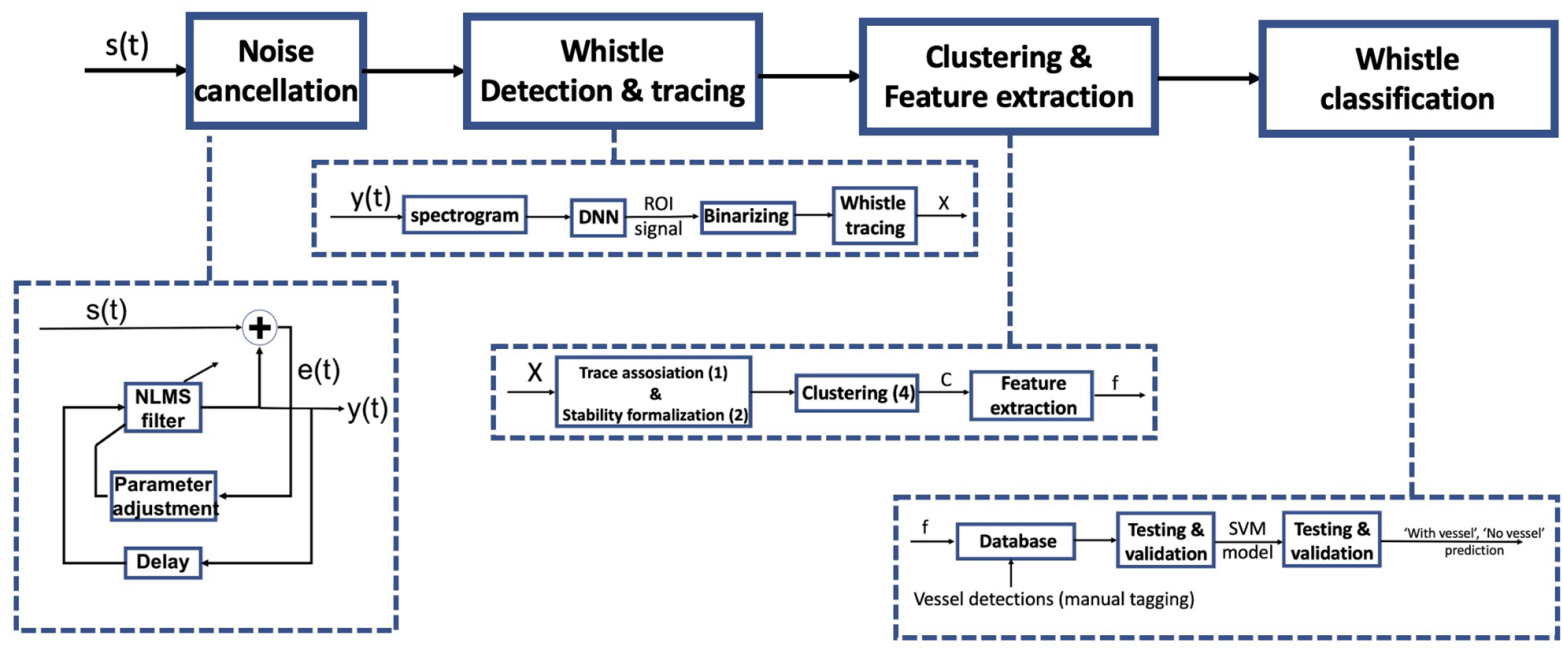
A block diagram of the methodology for dolphin’s whistles classification.

**Figure 11:**
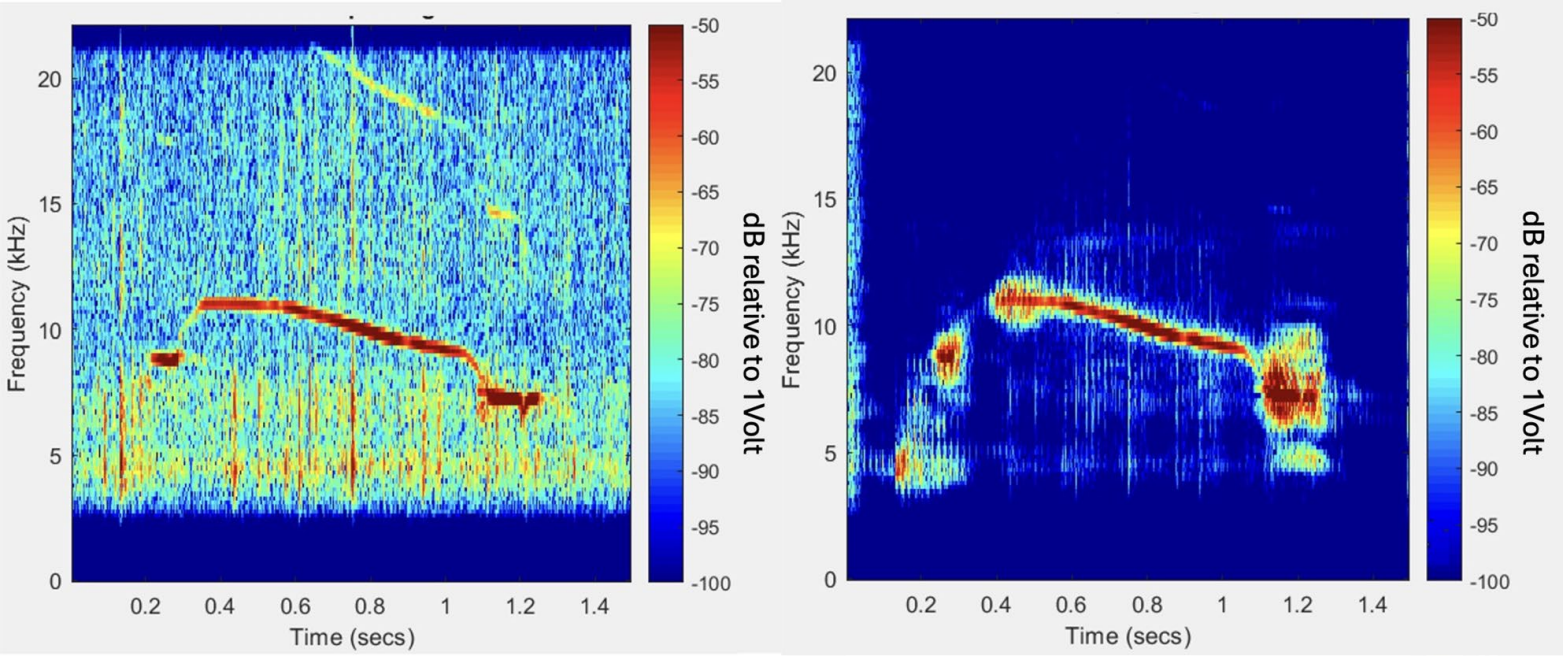
Example of the spectrum of a recorded dolphin whistle (left panel) and its noise cancellation version (right panel. SNR improvement is roughly 30 dB.

In this section, we define the methodological details of our analyses and discuss the algorithms used for noise cancellation, whistle detection, whistle clustering and whistle classification. A block diagram of our data analysis pipeline is given in Fig. 10. Our analysis starts with noise cancellation to improve the SNR. The process builds on the expected statistical dependency between the samples comprising the dolphin whistles to separate from the independent noise samples, and is performed by an adaptive normalized least mean square filter tuned to find the similarities between two signals recorded in consecutive time segments. Referring to the illustration in Fig. 10, the filter fits the delayed version of the input signal, *s*(*t*), such that the error signal, *e*(*t*), between the filtered signal and *s*(*t*) is minimized. The output of the filter, *y*(*t*), is then the desired signal with improved SNR. An example for the spectrogram of an input signal including a dolphin whistle and its noise cancellation version is shown in Fig. 11. Full details for the noise cancellation process are given in^26^.

### Whistle detection

A key step towards exploring the effect of vessel URN on dolphins is whistle detection. The time-varying characteristics of the dolphin whistles makes it favorable for detection by spectrum analysis. Previous approaches modeled the signal as a chirplet and used for detection the cross-correlation between the spectrum of the signal and a synthetic kernel representation of a whistle^27^, reporting that the “French hat” wavelet is a good fit for such kernel^28^. PAMGuard^29^, which is an open source passive acoustic monitoring (PAM) software, uses a flexible correlation method, which enables the user to build the correlation kernel segment-by-segment. Alongside this, PAMGuard offers an energy detector and a time-domain matched filter detector. Yet, the robustness of PAMGuard is limited to a dictionary of recorded sounds. To improve robustness, approaches motivated by speech recognition techniques exploited a pitch detection algorithm to identify harmonics in the signal’s spectrum and to estimate the pitch and its temporal changes^30^. These are then matched with a harmonic template that is less demanding than knowledge of the full chirplet signal. To detect sequences of whistles one can use a chain of peak energy detectors, a multi-band energy detector, and a spectral entropy detector, each designed to detect different features in the signal’s spectrum^31^. Others have proposed to use supervised neural networks to track frequency lines by assuming an underlying Markov model representation of the whistle’s curve^32^. Another general method to detect a signal of a distinguished spectrum pattern involves an edge detection filter to segment spectrum segments by fitting a quadratic equation to types of rising, falling, flat or blank spectrum shape^33^. However, the results obtained are below 80% for both the true positive and the true negative cases, which could create a bias in our analysis.

The design of an automatic whistle detection system confronts two main challenges. First, the signal is received within the ambient noise, which can be high such that the SNR is low. Second, the recorded signal may be affected by non-isotropic noise sources such as transient noises from snapping shrimps^34^ or depth meters and sonar signals, which makes it hard to establish and verify a desired false alarm rate. Our approach for detection thus begins with filtering the data between the 5 and 20 kHz band to capture most of the energy of the whistles^17^, followed with a wavelet de-noising filter^35^ to mitigate noise transients. Other noise sources were handled through an adaptive noise cancellation scheme^26^ that identifies signals by their samples’ dependencies.

After pre-processing, we performed whistle detection by a state-of-the-art deep neural network (DNN) carefully tagged by a human expert. We implemented a transfer learning network that is based on the VGG16 architecture that is suitable for object recognition^36^. To match with VGG, we converted the spectrogram image into 3D tensors and normalized the gray-scale intensities. For transfer learning, we replaced the top layers of the VGG with two fully connected layers with a ReLU activation function and an output layer based on a softmax activation and a binary cross-entropy as a loss function. The Adam optimizer was used to train the model, and hyperparameters were optimized using the Optuna framework^37^. Training involved 108,317 spectrograms, of which 49,807 were tagged as noise and 58,510 as dolphin whistles. Further details are given in^38^. After thresholding, the results of the DNN are segments of spectrogram images, termed *region of interest* (ROI) images, whose pixels are ranked by similarity to dolphin whistles traces.

To train the machine-learning whistle detector, we used manual tagging. Tagging was performed as part of a Citizen Science project, where high-school students from the ’Open School’ in Haifa, Israel, observed the created spectrograms and listened to the recorded data to mark the trace of identified whistles. For validation, tagging was performed separately by four different groups handling the same dataset. Disagreements among the groups were resolved by an expert. The students were given a full day training by a sonar expert. The students manually tagged parts of the acquired dataset, which was considered as a training dataset for the detector. We have randomly selected four hours from each day of recording. Following the custom four-fold approach, we have divided the training dataset into training, evaluation, and testing. To avoid overfitting, whistles in the testing part were never seen by the machine learning trainer.

### Whistle classification

**Figure 12:**
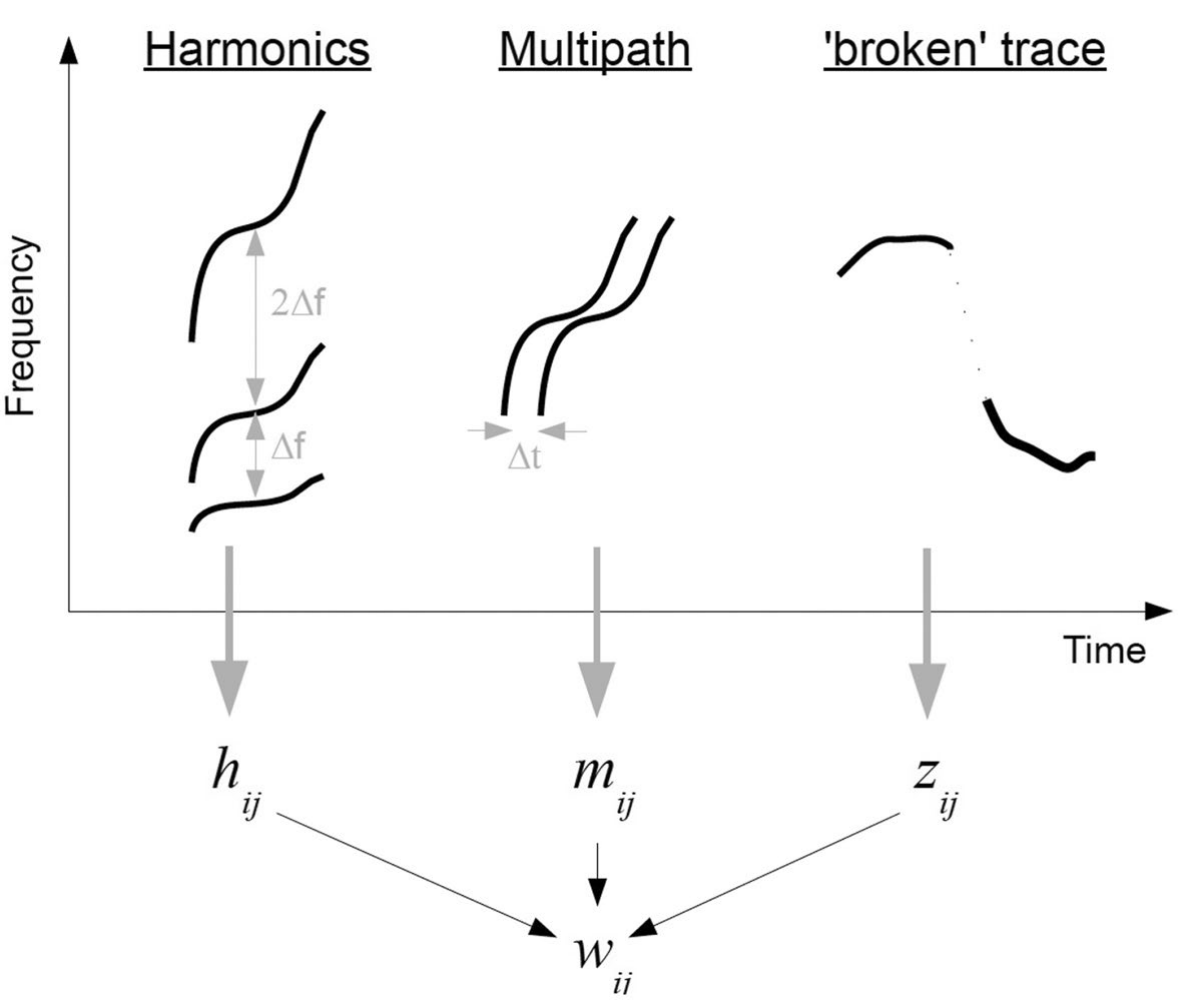
Illustration of the clustering procedure.

The classification of whistles into the two classes: “with vessel” and “no vessel” is based on feature analysis. To extract features such as the number of overlapping whistles or rate of harmonics, we performed a preliminary clustering procedure. Our process is illustrated in Fig. 12 and includes three steps: 1. discovery of all traces of the whistles; 2. identification of multi-path, harmonics and discontinuities in the trace of the whistles; and 3. solving an optimization problem to merge all identified traces into whistle groups. The last step labels the set of dolphin whistle traces, where each number represents a different whistle, and is stemmed from the likelihood of each trace to be connected to its nearby traces, both in the time and the spectral domains. The process is presented in detail elsewhere^39,40^ and its key idea is given here for completeness.

To identify the whistles traces, we identify curve-like patterns in the ROI image. These curves are a time-frequency representative of the whistle and its harmonics. While we avoid a hard assumption regarding the shape of the whistle, we do consider each the pixels of each time-frequency trace to be stationary, i.e., follow a statistical pattern that is different than that of the noise. Our aim is thus to identify the most probable sequence of pixels within the spectrogram image. Formally, let *c*(*i*, *j*) be the pixel value in the *i*th frequency bin and the *j*th time bin of the spectrogram image. Also let $C_j=\{c(1,j),\ldots ,c(I,j)\}, \quad j=1,\ldots ,J$, where *I* and *J* are the number of frequency and time bins in the ROI image. We consider the frequency bins as states, $s_1,\ldots ,s_I$, and sets $C_j$ as observations, and find the sequence of *J* states that represent a whistle trace. To that end, we choose the Viterbi algorithm^41^ as a dynamic programming approach. Here, we regard the DNN’s detection output samples corresponding to the pixels of the spectrogram image as a measure of likelihood to contract the emission probability of the elements in the sequence. Further, assuming a maximum *steepness* of the dolphin whistle curve, we set limitations on the frequency difference between the pixels as transition probabilities. That is, for any consecutive pair of frequency-time cell indices, (*i*, *j*) and $(n,j+1)$, that comprise a valid whistle trace, we assume $0\le |n-i|<\rho$, and we set $\rho =3$ bins based on our own manually tagged database. The outcome of the Viterbi algorithm are sequences of state traces, $X_k$, sorted by their accumulated likelihoods. Placing a threshold over these probabilities would determine the number of identified traces, *N*, within the spectrogram image. Note that identified traces within a single ROI image can originate from different dolphins. Our next step is thus to cluster the identified *N* traces into single whistles by associating them to three types of basic whistles, harmonics or multi-path, such that each trace uniquely belongs to a single cluster.

Clustering the traces is performed by weighting their likelihoods to the three types. Denote $w_{i,j}$ as the resulting likelihood of two traces $X_i, X_j$ to share the same cluster encoded within an affinity matrix $\textbf{W}$. Since two whistle-traces cannot be simultaneously harmonics and delayed templates of each other, harmonics and continuum of each other, or delayed templates and continuum of each other, we set $w_{i,j}$ to account for the most likely phenomena of the three. Define a cluster $C_k$ as a vector whose dimension corresponds to a different trace. Formally,

1 $$\begin{aligned} \mathbf{{c}}_k = (\omega _{1,k},\dots ,\omega _{N,k})^{\textrm{T}} , \end{aligned}$$

where $\omega _{i,k}$ is an indicator for the association of the *i*th trace $X_i$ to the *k*th cluster $C_k$. The *stability* of the clusters is formalized as

2 $$\begin{aligned} \sum _{k=1}^K C_k^{\textrm{T}} \textbf{W}C_k - \sum _{k=1}^K C_k^{\textrm{T}} \textbf{D}C_k, \end{aligned}$$

where $\textbf{D}$ is a diagonal matrix whose (*i*, *i*) entry is the sum of similarities of the *i*th whistle-trace to all other whistle-traces,

3 $$\begin{aligned} d_{i,i} = \sum _{j=1}^N w_{i,j}. \end{aligned}$$

In (2), we combine the inter-cluster stability (first term) with penalty for too-large clusters (second term). The solution for the trace association is found by solving

4 $$\begin{aligned} \begin{aligned} \mathop {\text {arg}\,\text {max}}\limits _{C_k} \sum _{k=1}^K C_k^{\textrm{T}} \mathbf{(W-D)} C_k, \\ \text {s.t. } C_k \perp C_l, \forall k \ne l. \end{aligned} \end{aligned}$$

Once (4) is solved, the feature extraction is performed per cluster. Duration is set as the length of the basic whistle in the cluster; ’Harmonic Rate’ is calculated as the multiplication from the basic whistle; ’Number of Overlaps’ is determined by overlapping traces belonging to different clusters; and ’Whistle Number’ is the number of traces comprising a cluster.

Recall our aim is to prove the relationship between a dolphin whistle and URN from nearby vessels. To that end, we classify the features of the 51,231 identified ROI images into “with vessel” and “no vessel” classes, and measure the classification success in terms of the TP and TN rates compared to the ground truth information. For classification we use a non-linear SVM with radial basis kernel. We choose SVM as a simple classification tool that works directly on the features of the signal, thereby allowing drawing conclusions of what are the important features for classification. We note that a classification attempt using a Long Short Term Memory (LSTM) network with sigmoid activation that works directly on the ROI image or on the whistle features (as a matrix of features) was less successful, probably because the reduced number of features increased the risk of overfitting for classifiers based on neural networks. Additional trials with K-NN and K-means classifiers indeed yielded better performance, but inferior to the SVM. We take a four-fold approach and determine our SVM model by dividing the database into train and validation phases. To avoid overfitting, instead of picking random whistles, we consider data obtained within a full day as independent and perform testing separately for each calendar day. That is, for a chosen day, we perform training and validation for all other days and testing only for the chosen day.

## Data Availability

The raw acoustic data, the identified dolphin whistles, and the tagging indications of vessels are available in^42^.
